# Surface frontogenesis by surface heat fluxes in the upstream Kuroshio Extension region

**DOI:** 10.1038/s41598-017-10268-3

**Published:** 2017-08-31

**Authors:** Tomoki Tozuka, Meghan F. Cronin, Hiroyuki Tomita

**Affiliations:** 1 Department of Earth and Planetary Science, Graduate School of Science, The University of Tokyo, Tokyo, Japan; 2 NOAA Pacific Marine Environmental Laboratory, Seattle, WA USA; 3 Institute for Space-Earth Environmental Research, Nagoya University, Nagoya, Japan

## Abstract

Western boundary currents bring warm tropical water poleward and eastward and are characterized by a sharp sea surface temperature (SST) front on the poleward edge of the current as it extends into the interior basin. One of the most prominent such front is associated with the Kuroshio Extension (KE) as it extends east of Japan (“upstream KE”). Large latent and sensible heat fluxes that warm the atmosphere and cool the ocean project this front into the atmosphere, thereby affecting weather and climate both locally and remotely. While one might assume that these larger surface heat fluxes on the equatorward side would tend to damp the SST front, here we present observational evidence that the surface heat loss actually strengthens the front during October-April in monthly climatology and about 87% of months from October to January during the 2004/05–2014/15 period, although the percentage lowers to about 38% for February-April of the same period, suggesting some temporal/data dependency in the analysis. The key to understanding this counterintuitive result for frontogenesis is that the effective heat capacity of the surface water depends on mixed layer thickness. SSTs are more (less) sensitive to surface heat fluxes in regions with shallow (deep) mixed layer.

## Introduction

**Figure 1:**
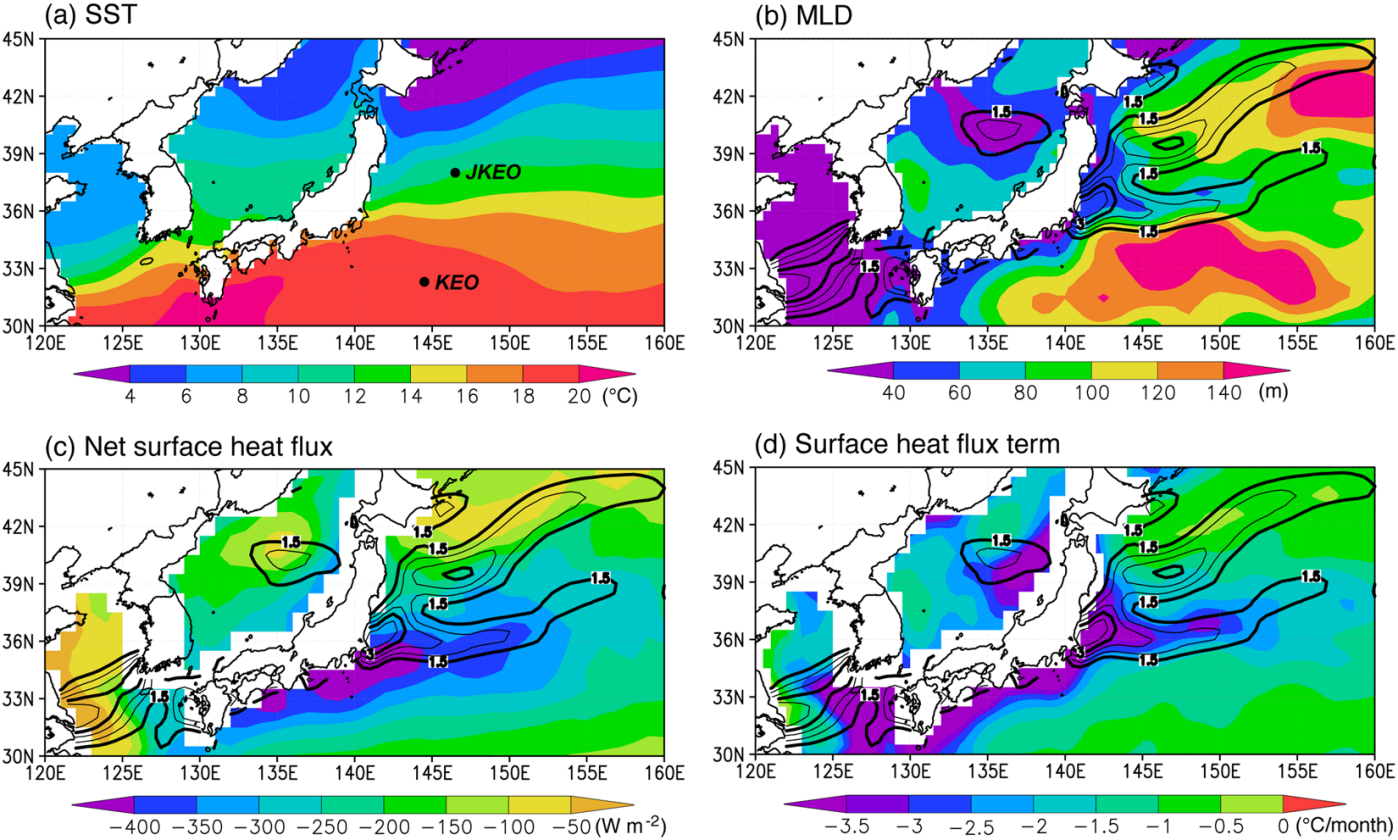
Winter (February) climatology. Monthly climatology of (**a**) sea surface temperature (SST) (in °C), (**b**) mixed layer depth (MLD) (in m), (**c**) net surface heat flux (in W m^−2^), and (**d**) the surface heat flux term in Eq. (1) (in °C month^−1^) in February. The KEO and JKEO mooring sites are shown in (**a**) and absolute values of the meridional SST gradient (in °C °latitude^−1^) are shown in (**b**–**d**) with contours. The contour interval is 1.5 (0.5) °C °latitude^−1^ for thick (thin) contours and contours less than 1.5 °C °latitude^−1^ are not drawn. The figure was prepared with GrADS v.1.9b4 (http://cola.gmu.edu/grads/).

A strong sea surface temperature (SST) front exists in the Kuroshio Extension (KE) region off the east coast of Japan^1–3^ associated with the northwestern branch of the North Pacific’s subtropical gyre. As shown in Fig. 1a, SST decreases drastically northward from 18 °C at 34°N to 12 °C at 38°N in the 145°E–150°E band in February, when the SST front is at its seasonal maximum in the mid-winter. The strong SST meridional gradient in the 145°E–150°E band (referred to as the “upstream KE”)^4^ is associated with the northern branch of the North Pacific’s subtropical recirculation gyre. A high SST meridional gradient north of the KE shown in Fig. 1 is associated with the Oyashio Extension of the subarctic gyre. In this study, we focus on processes affecting the upstream KE SST front.

The KE SST front has been shown to play an important role in the active air-sea interaction in the northwestern Pacific^5^. Based on mooring observations on both sides of the SST front, Konda *et al*.^6^ showed that the front has profound impacts on latent and sensible heat fluxes depending on wind conditions. Using atmospheric models with and without the sharp SST front, past studies^7–9^ found strong impacts of the front on the wintertime surface turbulent heat fluxes and storm-track activity through the so-called oceanic baroclinic adjustment^1, 10, 11^. The KE SST front also has been observed to influence the vertical development of clouds^12, 13^. During early summer, sea fog frequently forms on the northern flank of the front as the warm southerly wind blows over the cool ocean. In contrast, during winter, as cool northerly winds blow across the front, strong heating of the marine atmospheric boundary layer by turbulent heat fluxes on the southern (warm) flank of the front lead to enhanced frontogenesis in the atmosphere, with lowered sea level pressure^14^ and anomalous surface wind convergence^15^ over the warm water. As a result, the cloud top on the southern flank penetrated above the marine atmospheric boundary layer, reaching the mid-troposphere. The destabilization of the marine atmospheric boundary layer over the warm SST also results in higher wind speed compared with areas with colder SST^16^. In addition to these seasonal variations, decadal variations of the SST front are suggested to play an important role in the North Pacific climate variability^17^.

**Figure 2:**
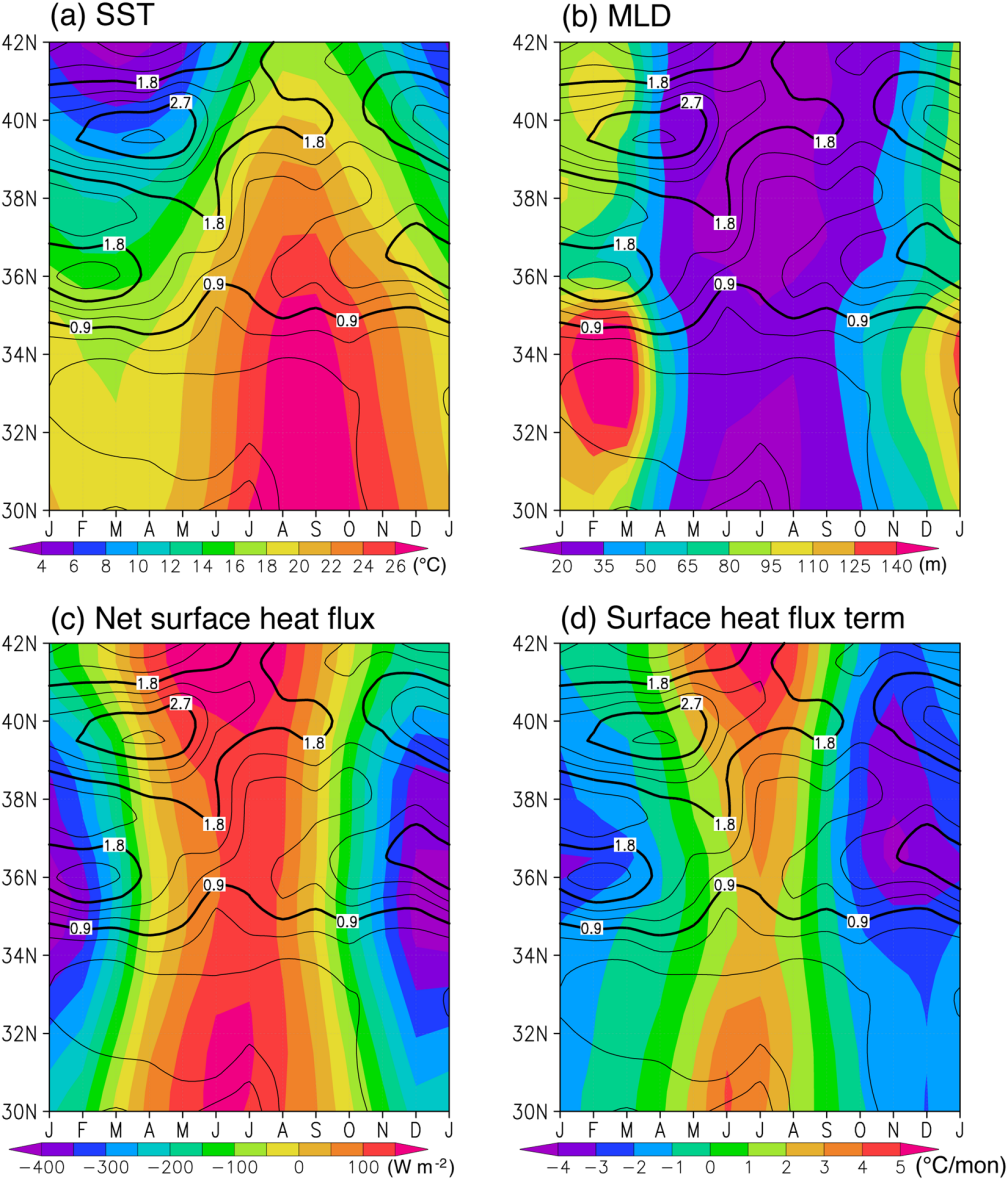
Seasonal variation in SST, MLD, net surface heat flux, and surface heat flux term. Latitude-time plots of (**a**) SST (in °C), (**b**) MLD (in m), (**c**) net surface heat flux (in W m^−2^), and (**d**) surface heat flux term in Eq. (1) (in °C month^−1^) for the 145°E–150°E band. Also drawn are absolute values of the meridional SST gradient (contours, in °C °latitude^−1^). The contour interval is 0.9 (0.3) °C °latitude^−1^ for thick (thin) contours. The figure was prepared with GrADS v.1.9b4 (http://cola.gmu.edu/grads/).

Although the importance of the KE SST front is well recognized^18^, few studies have questioned how the front is maintained and evolves. Figure 2a shows a time-latitude plot of the meridional SST gradient for the 145°E–150°E band. During boreal winter, the meridional SST gradient has two maxima; one associated with the KE at around 36°N with a maximum of about 2.2 °C/°latitude in February and the other associated with the subarctic front at around 39.5°N with a maximum of 3.0 °C/°latitude in April. Although the latter exists throughout the year, the former, which is the main focus of this study, is conspicuous only from boreal autumn to spring with the maximum in February. The seasonal variation in the meridional SST gradient across the KE is in agreement with that obtained by Kida *et al*.^18^ with the Merged satellite and *in situ* data Global Daily SST (MGDSST)^19^ and the Japanese Fishery Research Agency-Japan Coastal Ocean Prediction Experiment (FRA-JCOPE2) reanalysis data^20^. Although one might assume that heat advection by the strong oceanic current maintains the SST front and that large surface heat loss over the southern flank of the SST front tends to damp this front, no quantitative support to this notion has been provided. Here, we investigate quantitatively the mechanism for the mid-winter strengthening of the SST front in the upstream KE.

## Results

**Figure 3:**
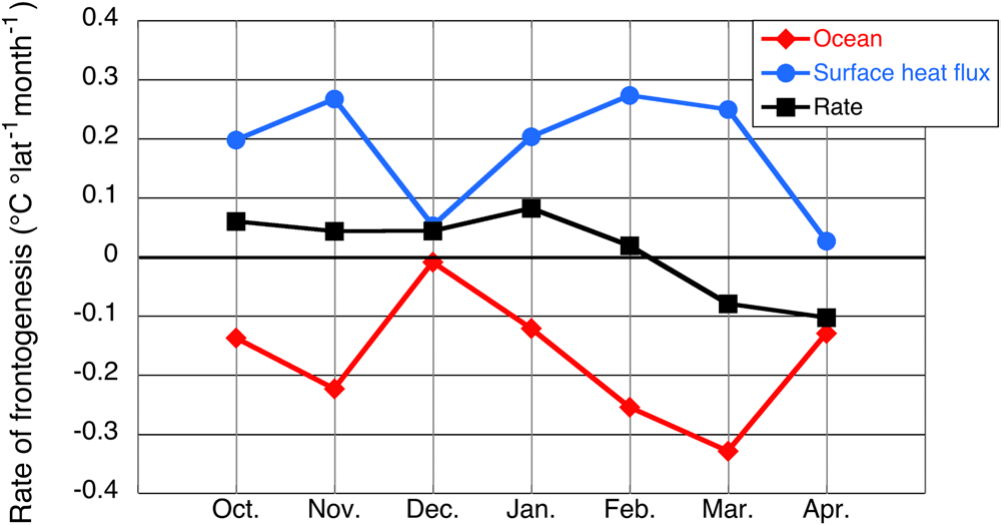
Seasonal variation in the rate of frontogenesis. Contribution of oceanic (red; the second term on the right hand side of Eq. (3)) and surface heat flux (blue; the first term on the right hand side of Eq. (3)) terms to the frontogenesis (black; the left hand side of Eq. (3)) from October to April at 145°E–150°E (in °C °latitude^−1^ month^−1^). A positive (negative) rate of frontogenesis indicates frontogenesis (frontolysis). The figure was prepared with Kaleida Graph 4.0.

To understand how the very large seasonal surface heat loss over the warm KE water affects the strength of the SST front, we calculate the surface heat flux and oceanic terms in the frontogenesis/frontolysis equation (3). To estimate the meridional gradients across the front, we compute the difference between the 5° longitude × 3° latitude boxes on both sides of the front (Fig. 3) (see Methods). We limit our analysis to the periods from October-April because the SST front in the longitude band 145°E–150°E becomes conspicuous during boreal autumn to spring (Fig. 2); at other times, it is difficult to define the boxes on both sides of the SST front. The rate of frontogenesis is positive (negative) and the SST front is strengthening (relaxing) from October to February (March to April). Intriguingly, the surface heat flux term contributes to the frontogenesis from October to April, while the oceanic term as a whole contributes to the frontolysis from October through April.

To determine why the surface heat flux term strengthens the SST front, we examine the time-latitude plots of the mixed layer depth (MLD) and the net surface heat flux for the 145°E-150°E band (Fig. 2b–c). The net surface heat flux is strongly negative in winter because cold and dry air from the continent blows across the warm KE^6^ and there is a strong eddy-driven westerly jet associated with the SST front^1, 6, 10^; the maximum cooling of over 400 W m^−2^ exists around and south of the SST front (Figs 1c and 2c). We note that on synoptic times-scale, surface heat loss to the atmosphere is sometimes greater on the poleward side of the SST fronts^6, 21^, but on the monthly time-scale, which is the focus of this study, the surface heat loss is generally greater on the equatorward side of the SST front. Thus, if the MLD were constant across the SST front, the surface heat flux is expected to relax the SST front. However, large seasonal and meridional variations are present in the MLD (Figs 1b and 2b). In general, the MLD is deeper to the south of the SST front and the deepest mixed layer of over 150 m is found around 33.5°N in February, while there exist local minima in the MLD just to the north of the SST front. The shallower (deeper) mixed layer to the north (south) makes it more (less) sensitive to the wintertime cooling by the surface heat flux and this is why the surface heat flux term contributes to the frontogenesis in boreal winter; the maximum cooling by the surface heat flux term reaches −4.0 °C month^−1^ just north of the SST front (Figs 1d and 2d).

**Figure 4:**
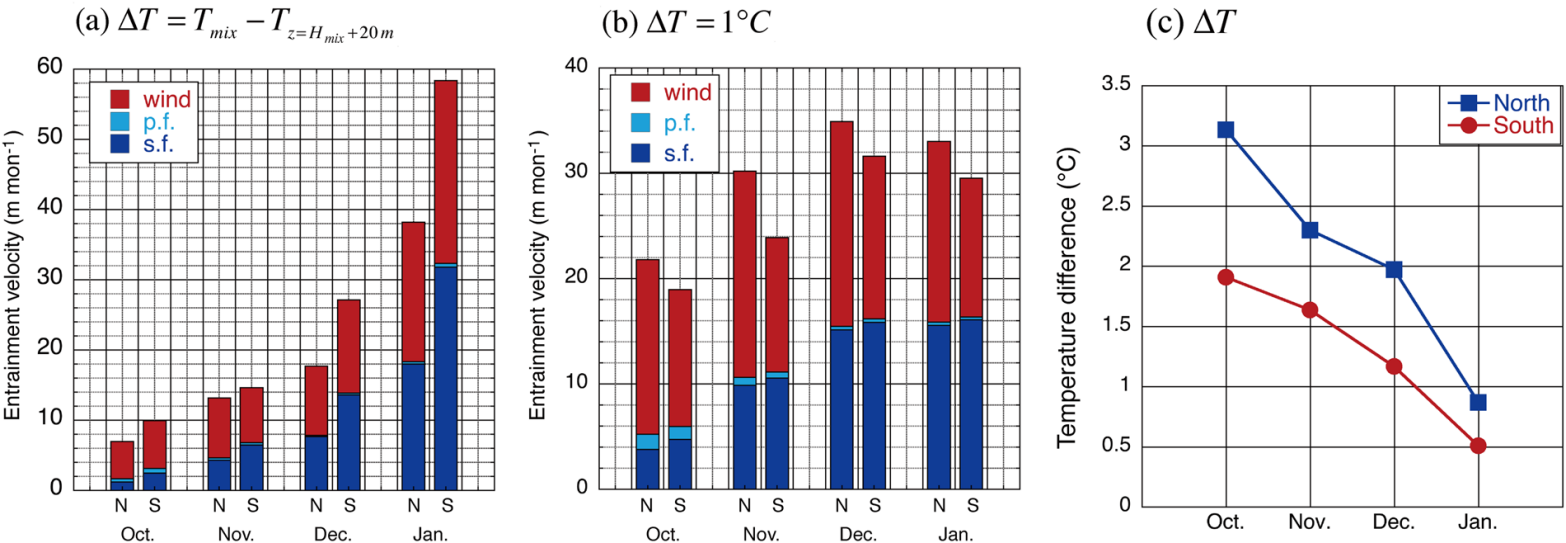
Entrainment velocity on both sides of the SST front. (**a**) Monthly climatology of the entrainment velocity (m month^−1^) calculated by Eq. (4) from October to January and its contributing terms in the northern (N) and southern (S) boxes. Here, “wind” (red bar) denotes the wind stirring term (the first term on the right hand side of Eq. (4)), “p.f.” (light blue bar) denotes the radiative penetration flux term (the second term on the right hand side of Eq. (4)), and “s.f” (blue bar) denotes the surface buoyancy flux term (the third term on the right hand side of Eq. (4)). (**b**) As in (**a**), but for monthly climatology of entrainment velocity calculated with a constant temperature difference between the mixed layer and the entrained water (Δ*T* = 1 °C). (**c**) Monthly climatology of the temperature difference (in °C) between the mixed layer and the entrained water (Δ*T*) on the northern (blue) and southern (red) sides of the SST front. The figure was prepared with Kaleida Graph 4.0.

This raises the following question: what causes the shallower (deeper) mixed layer to the north (south) of the SST front? We answer this question by calculating the entrainment velocity using eq. (4) (see Methods). The entrainment velocity is larger to the south from October to January, and the difference is particularly large in December and January (Fig. 4a). Both the surface heat flux and wind stirring effects have large contributions, but the surface heat flux effect contributes more to the larger entrainment velocity to the south in both months. On the other hand, the penetrative flux effect is negligible throughout the deepening phase.

The larger entrainment velocity on the equatorward side is due to the smaller difference between the mixed layer temperature and entrained water (Fig. 4c) owing to the deeper thermocline. Because more energy is required to entrain colder and heavier water, the entrainment velocity tends to be smaller to the north of the SST front. In fact, if a constant temperature difference of Δ*T* = 1 °C is used for both sides of the front, the entrainment velocity is slightly larger on the poleward side (Fig. 4b) due to a stronger wind stirring effect.

In contrast to the surface heat flux term, the oceanic term as a whole acts to reduce the meridional SST gradient. Because this is estimated as a residual (it is not possible for us to estimate the oceanic terms with adequate precision using available observation data), horizontal and vertical oceanic processes cannot be distinguished. However, scale analysis shows that the horizontal diffusion may be order one. For example, if we assume a constant horizontal eddy diffusivity of 1.0 × 10^3^ m^2^ s^−1^ ^22^, the rate of frontogenesis by the horizontal diffusion in January is estimated to be −0.4 °C °latitude^−1^ month^−1^. This is roughly comparable to the frontolysis by the oceanic terms. Thus, the horizontal mixing may provide the dominant mechanism for the frontolysis. A more detailed study of the oceanic terms in the surface frontogenesis/frontolysis is underway that uses a high-resolution ocean general circulation model to distinguish the contributions from horizontal advection, horizontal diffusion, and vertical processes.

**Figure 5:**
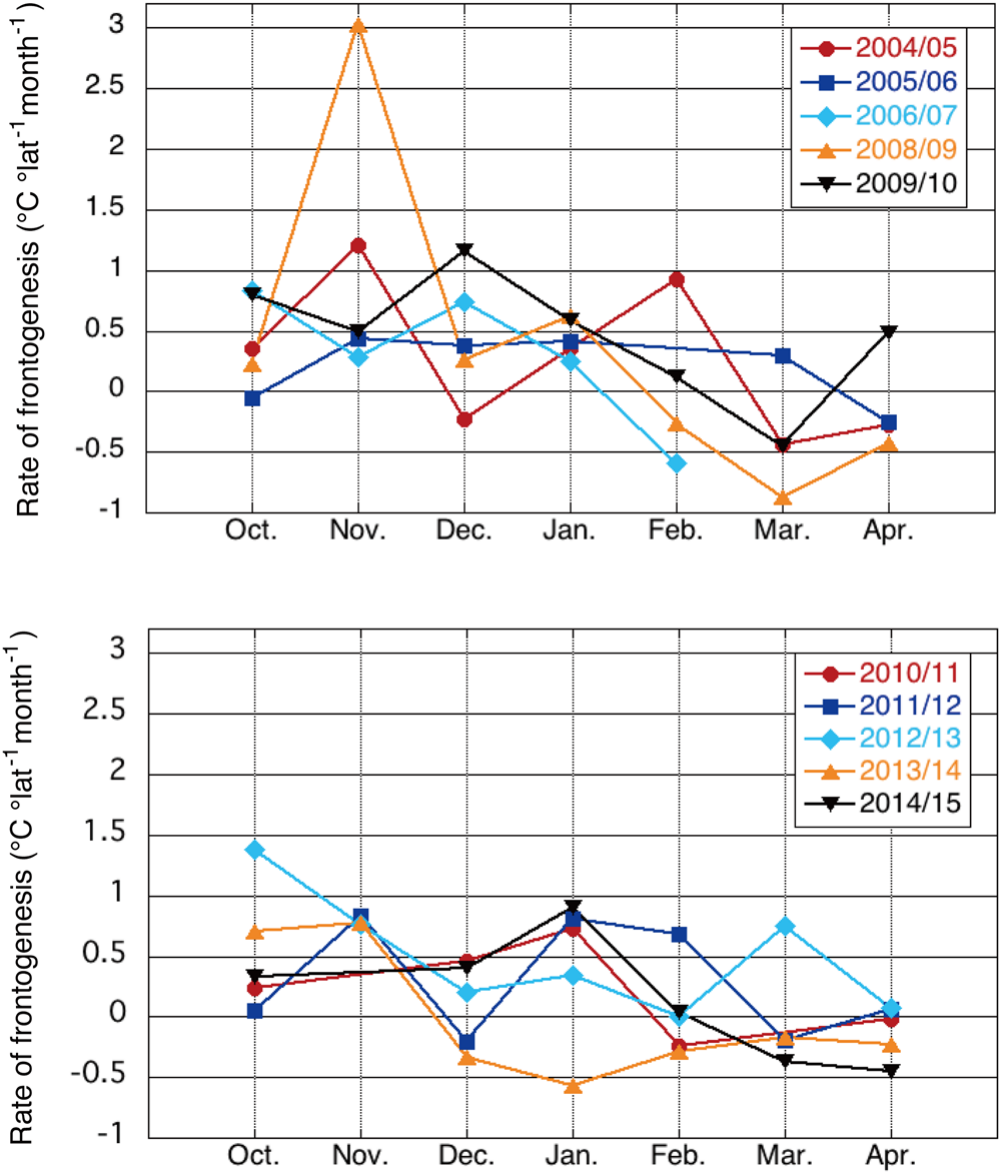
Rate of frontogenesis by the surface heat flux term from 2004/05 to 2014/15. Contribution of the surface heat flux terms to the frontogenesis (the first term on the right hand side of Eq. (3)) from 2004/05 to 2014/15 for October-April at 145°E–150°E (in °C °latitude^−1^ month^−1^). A positive (negative) rate of frontogenesis indicates frontogenesis (frontolysis). Since it was not possible to define the SST front in the KE region for three months, 2007/08 is not plotted. Also, the SST front was not identifiable in March-April 2007. The figure was prepared with Kaleida Graph 4.5.

Since the above analyses used climatological datasets, the year-to-year variations in these processes are investigated by considering their monthly time series from 2004 to 2015 of the datasets described in Methods. This will also allow us to look at the effects of meandering and meso-scale eddy activity on the monthly time-scale. As shown in Fig. 5, the surface heat flux term tends to strengthen the SST front in most years from October to January, although the surface heat flux term tends to relax the SST front in many years after February. More specifically, the surface heat flux term reinforces the SST front in about 87% of the years from autumn to early winter (October-January), while the percentage lowers to about 38% from late winter to early spring (February-April). The lower percentage compared with the climatological analyses for February-April (see Fig. 3) may be due to the different temperature and salinity data (see Methods section) used for the climatological (Fig. 3) and interannual (Fig. 5) analyses. The former has a horizontal resolution of 0.5° × 0.5°, while the latter has a horizontal resolution of 1° × 1°. Also, the weighting and covariance functions used to prepare the former data are designed to sharpen front. These may be why the MLD gradient across the KE SST front in the climatological data is stronger than that in the month-to-month data, and results in a more frontogenetic contribution by the surface heat flux term in the climatological analysis. Weaker climatological meridional MLD gradient in the month-to-month data in February-April may explain the lower percentage from late winter to early spring. We also note that the skewness of the surface heat flux term is 0.78, indicating that the frontogenesis producing values are greater than the frontolysis producing values. In particular, when the average rate of frontogenesis by the surface heat flux term is calculated, the average rate is positive for February. However, the average rate is negative for March and April.

**Figure 6:**
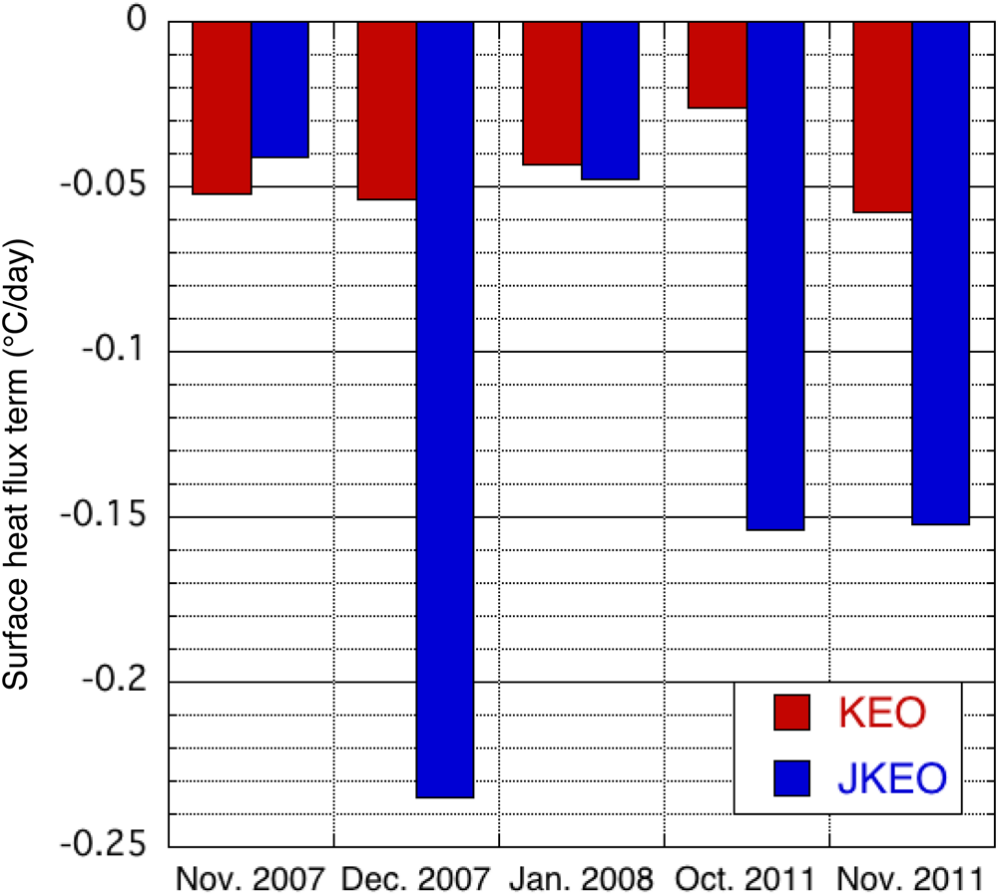
Surface heat flux term estimated with mooring data. Surface heat flux term (the first term on the right hand side of Eq. (1); in °C day^−1^) at the KEO (red bar) and JKEO (blue bar) mooring sites. The location of the mooring sites is shown in Fig. 1a. The figure was prepared with Kaleida Graph 4.0.

To give further confidence that the above features seen in the gridded data sets are robust results, we perform a similar analysis using mooring observations. In particular, we have estimated the surface heat flux term obtained from two moorings located on the northern and southern sides of the KE front^6, 23, 24^. Except for one month, cooling by the surface heat flux term was greater on the northern side (JKEO mooring) (Fig. 6), in agreement with our results obtained from the gridded data.

## Discussion

The fact that the surface heat flux actually strengthens the SST front in the upstream KE region, particularly from autumn to early winter, is a remarkable result that has not been seen in other frontal regions. For instance, surface heat fluxes tend to weaken the SST front in the Agulhas Return Current region from 40°E to 55°E^25, 26^, although the meridional variations in MLD do affect the sensitivity of the ocean to the surface heat flux. The frontolysis is amplified (damped) in austral summer (winter), because warming (cooling) by the surface heat flux is amplified south of the front where the MLD is shallower, and is reduced north of the front where the MLD is deeper. The difference between these two frontal regions may stem from the smaller meridional gradient of the net surface heat flux across the KE front compared with that across the Agulhas Return Current front; the latter is located at 42.5°S in the 45°–50°E band in July and the net surface heat flux is −101 W m^−2^ (4 W m^−2^) when averaged over the 5° longitude × 3° latitude box on the equatorward (poleward) side of the front^25^. Thus, the unique location of the KE SST front under the influence of the Asian winter monsoon may be the reason why the surface heat flux term contributes to the frontogenesis and makes the mid-winter SST front so conspicuous. Since the SST front is obscure in summer (Fig. 2a), it is difficult to define boxes on both sides of the SST front and perform the same quantitative analysis in summer. However, the surface heat flux term tends to reduce the meridional SST gradient, because the net surface heat flux into the ocean is larger on the northern side of the SST front and deeper (shallower) mixed layer to the south (north) of the SST front is less (more) sensitive to the strong surface heating in summer.

Our analysis has used monthly-averaged time series and climatologies to evaluate the frontogenesis/frontolysis processes in the upstream KE region. In the KE region, it has been shown that meso-scale air - sea heat fluxes play an important role^27, 28^; heat release to the atmosphere is enhanced over warm eddies north of the SST front^29^. This means that meso-scale air-sea heat flux would tend to increase surface heat loss to the north of the SST front, and decrease the meridional gradient in surface heat fluxes across the SST front. This is favorable for the surface heat flux term to act as a frontogenesis mechanism. The mixed layer, however, may be deeper in the vicinity of warm eddies to the north, and such effects may reduce the meridional MLD gradient. Since the meridional MLD gradient is weaker in late winter, this may partially explain why the surface heat flux term tends to reinforce the SST front from October to January in about 87% of months, while the percentage lowers to about 38% in February-April. In fact, for about 90% of the months where the surface heat flux term relaxes the SST front (19 months out of 21 months), the meridional MLD gradient is smaller than the climatology or the sign of the meridional gradient is actually reversed. Mesoscale eddies seem to contribute to this smaller or reversed MLD gradient. The role of meso-scale eddies in the processes discussed in this study is an interesting research topic and will be investigated in more detail in the future when model outputs of the High Resolution Model Intercomparison Project (HighResMIP)^30^ becomes available.

An important implication of this study is that for coupled general circulation models to properly reproduce frontal air-sea interaction effects, they need to have realistic ocean mixed layers. Also, the warming rate at the subtropical western boundary currents and their extensions, which are partly associated with the meridional shifts in the oceanic fronts, are two to three times faster than the global mean warming^31^. The results presented in this study may serve to provide an additional factor that need to be considered when evaluating climate models and discussing warming in key regions of the global climate system.

## Methods

### Observational data

We use temperature and MLD data from the Monthly Isopycnal/Mixed-layer Ocean Climatology (MIMOC) data^32^. This data has 0.5° × 0.5° resolution and is based primarily upon Argo float profiles during the 2007–2011 period, but supplemented with historical CTD data. For the surface heat flux data, the Japanese ocean flux data sets with use of remote sensing observations (J-OFURO2) data^24^ is used, but we note that we obtain qualitatively the same results even if we use the objectively analyzed air-sea flux (OAFLUX) data^33^. The latter is used for the entrainment velocity calculation. Their horizontal resolution is 1° × 1°. We use data from 2000 to 2009 to calculate its monthly mean climatology, and negative values mean that the ocean loses heat. For interannual data analyses from 2004 to 2015 shown in Fig. 5, the JRA-55 reanalysis data^34^ is used for surface heat fluxes and for mixed layer depth, temperature and salinity data based on Argo floats prepared by Roemmich and Gilson^35^ are used. The former is on T319 grid (about 0.6° resolution in the meridional direction near the SST front in the Kuroshio Extension region), while the horizontal resolution is 1° × 1° for the latter. The former is interpolated to the latter grid when calculating the surface heat flux term.

Data from the Kuroshio Extension Observatory (KEO) and Japan Agency for Marine-Earth Science and Technology (JAMSTEC) Kuroshio Extension Observatory (JKEO) mooring are used^6, 23, 24^. The KEO mooring was deployed at 144.5°E, 32.3°N, while JKEO was deployed at 146.5°E, 38.0°N. Data during November 2007-January 2008 and October-November 2011, when both moorings had winter data, are used in this study. We note that the MLD for the mooring observations is defined as the depth at which the density increases by 0.03 kg m^−3^ from density at 10 m depth to avoid warm diurnal stratification contamination of the ocean mixed layer.

### Defining the SST front and boxes on both sides of the front

In this study, the SST front is defined as the maximum in the meridional SST gradient, and it is located at 36.5°N during October-December and 36.0°N during January-April. Qualitative results remain the same even if we use mixed layer temperatures to define the front.

For mixed layer temperature balance analyses, we use 5° longitude × 3° latitude boxes on both sides of the front. Since our analysis may be influenced by meandering, we have checked how much the SST front shifts meridionally by calculating the standard deviation of the latitudinal position of the SST front using the AMSR-E SST dataset with a horizontal resolution of 0.25° during 2003–2010. Here, the KE SST front is defined by the southernmost maximum of the absolute value of the meridional SST gradient greater than 1.8 °C/°latitude between 32°N and 39°N. It is found that the standard deviation is 0.80°, suggesting that it is reasonable to use boxes with the meridional extent of 3°. Thus, we have decided to use 3° latitude boxes so that the effect of the change in position of the actual KE front can be suppressed, although the qualitative results remain almost the same even if we use smaller 5° longitude × 1° latitude boxes.

### Mixed layer temperature balance and entrainment velocity

Since the SST is almost equivalent with mixed layer temperature, a calculation of mixed layer temperature balance^36, 37^

1 $$\begin{array}{rcl}\frac{\partial {T}_{mix}}{\partial t} & = & \frac{{Q}_{net}-{q}_{d}}{{\rho }_{o}{C}_{p}{H}_{mix}}-[{\vec{u}}_{mix}\cdot \nabla {T}_{mix}+\frac{1}{{H}_{mix}}\nabla \cdot {\int }_{-{H}_{mix}}^{0}\,\widehat{\vec{u}}\hat{T}dz]-\nabla \cdot (\kappa \nabla {T}_{mix})+(vertical)\\  & = & \frac{Q}{{\rho }_{o}{C}_{p}{H}_{mix}}+(oceanic)\end{array}$$

provides useful information about what processes determine the SST. Here, *T*_*mix*_ is the temperature averaged over the mixed layer, *H*_*mix*_ is the MLD, ${\rho }_{o}$ and ${C}_{p}$ are the density and specific heat of the sea water, respectively, ${\vec{u}}_{mix}$ is the horizontal velocity averaged over the mixed layer, $\hat{T}$ and $\widehat{\vec{u}}$ are the deviations from the vertically averaged temperature and horizontal velocity over the mixed layer, respectively, *κ* is the horizontal diffusion coefficient, ${Q}_{net}$ is the net surface heat flux into the ocean, ${q}_{d}$ is the penetrative shortwave radiation at the mixed layer base, $Q={Q}_{net}-{q}_{d},(vertical)$ is the vertical diabatic term that includes vertical diffusion and entrainment, and $(oceanic)$ is the sum of all oceanic terms. The downward shortwave radiation at depth *z* is parameterized^38^ by

2 $$q(z)=q(0)[Rexp\frac{z}{{\gamma }_{1}}+(1-R)exp\frac{z}{{\gamma }_{2}}],$$

where *R* (=0.58) is a separation constant, and ${\gamma }_{1}$ (=0.35 m) and ${\gamma }_{2}$ (=23.0 m) are attenuation length scales^39^. Taking the meridional derivative of Eq. (1), we may obtain the rate of frontogenesis/frontolysis^25, 26, 40^:

3 $$\frac{{\rm{\partial }}}{{\rm{\partial }}t}(\frac{{\rm{\partial }}{T}_{mix}}{{\rm{\partial }}y})=\frac{{\rm{\partial }}}{{\rm{\partial }}y}(\frac{Q}{{\rho }_{o}{C}_{p}{H}_{mix}})+\frac{{\rm{\partial }}}{{\rm{\partial }}y}(oceanic).$$

Here, the monthly mean climatology from the MIMOC data is used for the mixed layer temperature and depth, that from the J-OFURO2 data is used for the surface heat flux, and the second term on the right hand side is computed as a residual.

When only one-dimensional effects are considered, entrainment velocity at the base of the mixed layer during the deepening phase of the MLD with a negative net surface heat flux may be calculated using the Niiler and Kraus model^41^ by

4 $${w}_{e}=\frac{2\,{m}_{o}{{u}_{s}}^{3}}{\alpha g{H}_{mix}{\rm{\Delta }}T}+\frac{2{\int }_{-{H}_{mix}}^{o}\,q(z)dz}{{\rho }_{o}{C}_{p}{H}_{mix}{\rm{\Delta }}T}-\frac{(1-{m}_{c}){Q}_{net}+{q}_{d}}{{\rho }_{o}{C}_{p}{\rm{\Delta }}T}.$$

Here, $\alpha$ is the thermal expansion coefficient, *g* is the acceleration due to gravity, ${\rm{\Delta }}T$ is the temperature difference between the mixed layer and 20 m below the mixed layer base^37^, ${u}_{s}$ is the frictional velocity, ${m}_{o}$ is the efficiency coefficient of the wind stirring effect, which is taken to be 0.5 following Davis *et al*.^42^, and ${m}_{c}$ is the convective efficiency coefficient, which is taken to be 0.83 following Deardorff *et al*.^43^. We use the MIMOC data to obtain ${\rm{\Delta }}T$ and ${H}_{mix}$, and the monthly climatology of the OAFLUX data for wind speed and surface heat fluxes. We note that our results regarding the entrainment velocity are not so sensitive to the definition of ${\rm{\Delta }}T$; we obtained qualitatively the same results even if we use 10 m instead of 20 m. The three terms on the right hand side of Eq. (4) represent wind stirring, penetrative shortwave radiation, and surface buoyancy flux effects.

## Electronic supplementary material

Supplementary information
