# Supplementary material for: Surface frontogenesis by surface heat fluxes in the upstream Kuroshio Extension region
Source: Sci Rep. 2017 Aug 31;7:10258. doi: 10.1038/s41598-017-10268-3 (PMC5579054; doi:10.1038/s41598-017-10268-3)
Supplement: Supplementary file 1 — Supplementary information [file 41598_2017_10268_MOESM1_ESM.pdf]

Supplementary information for

"Surface frontogenesis by surface heat fluxes in the upstream Kuroshio Extension region"

Tomoki Tozuka[1, *], Meghan F. Cronin[2], and Hiroyuki Tomita[3]

[1] *Department of Earth and Planetary Science, Graduate School of Science,*

The University of Tokyo, Tokyo, Japan

[2] *NOAA Pacific Marine Environmental Laboratory, Seattle, WA, USA*

[3] *Institute for Space-Earth Environmental Research, Nagoya University, Nagoya, Japan*

**Corresponding author address: Dr. Tomoki Tozuka, Department of Earth and Planetary Science, Graduate School of Science, The University of Tokyo, 7-3-1 Hongo, Bunkyo-ku, Tokyo 113-0033, Japan. E-mail: tozuka@eps.s.u-tokyo.ac.jp*

Supplementary Figures

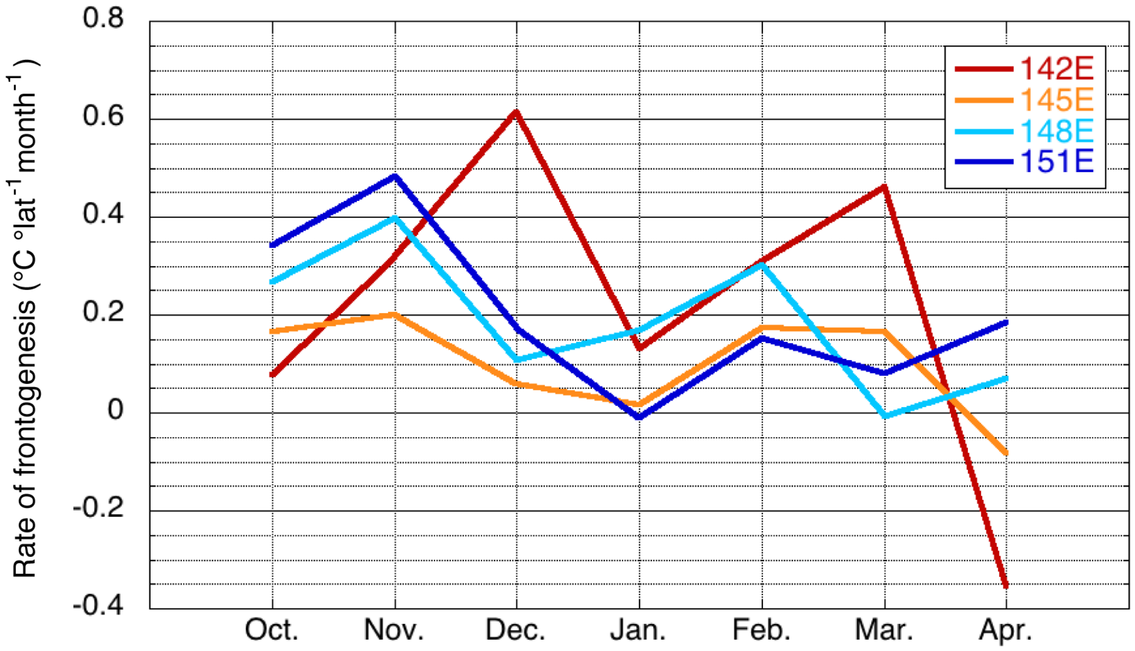

Figure S1: Seasonal variation in rate of frontogenesis by surface heat flux term in different longitude. Contribution of the surface heat flux term to the frontogenesis (the first term on the right hand side of Eq. (3); in °C °lat^{-1} month^{-1}) from October to April at 142°E (red line), 145°E (orange line), 148°E (light blue line), and 151°E (blue line). A positive (negative) rate of frontogenesis indicates frontogenesis. The figure was prepared with Kaleida Graph 4.0.
